# Microstructure and Mechanical Properties of Nb_35_Zr_26_Ti_19_Hf_15_Mo_5_ Refractory High-Entropy Alloy Under Rolling and Heat Treatment

**DOI:** 10.3390/ma18071643

**Published:** 2025-04-03

**Authors:** Hanjun Zhang, Baohong Zhu, Wei Jiang, Haochen Qiu, Shuaishuai Wu, Xuehui Yan, Shengli Guo

**Affiliations:** 1 State Key Laboratory of Nonferrous Structural Materials, China GRINM Group Co., Ltd., Beijing 100088, China; zhanghanjun7549@163.com (H.Z.); jiangwei@grinm.com (W.J.); qiuhaochen@grinm.com (H.Q.); wushuaishuai@grinm.com (S.W.); yanxuehui@grinm.com (X.Y.); guoshengli@grinm.com (S.G.); 2 GRIMAT Engineering Institute Co., Ltd., Beijing 101407, China; 3 General Research Institute for Nonferrous Metals, Beijing 100088, China

**Keywords:** high-entropy alloy, rolling, heat treatment, microstructure, mechanical properties

## Abstract

Refractory high-entropy alloys (RHEAs) have drawn much attention in the field of materials science for their unique properties and wide compositional design space. The Nb_35_Zr_26_Ti_19_Hf_15_Mo_5_ alloy is important for exploring RHEAs’ potential in high-temperature applications. It can break through existing material limitations and bring benefits to related fields, especially in the aerospace field. This paper focuses on Nb_35_Zr_26_Ti_19_Hf_15_Mo_5_ RHEAs and studies the effects of cold rolling and heat treatment on its microstructure and mechanical properties. The alloy has a single-phase BCC structure. As rolling reduction rises from 20% to 80%, the alloy’s strength increases notably while plasticity drops. At 80% rolling reduction, the tensile strength reaches 1408 MPa, and the elongation is 10.5%. During rolling, grains deform along the rolling direction, the number of low-angle grain boundaries grows, and dislocation and solid solution strengthening effects are enhanced. With the increase in annealing temperature, recrystallized grains increase, and the change in grain-boundary structure weakens the strengthening effect, leading to a strength decrease and a plasticity increase. After annealing at 800 °C, the elongation reaches 17%, and the dislocation density in the alloy decreases with a recrystallization degree of 49%.

## 1. Introduction

High-entropy alloys (HEAs) possess unique alloy design concepts and high-entropy effects, endowing them with potential application values in numerous fields. Unlike traditional alloys, HEAs are typically composed of four or more elements [1]. Currently, multi-principal-element HEAs have been summarized as having four major effects: the high-entropy effect in thermodynamics, which interferes with the formation of complex phases; the lattice distortion effect in structure, where severe lattice distortion can impede dislocation motion to some extent and enhance the alloy’s strength [2,3]; the sluggish diffusion effect in kinetics, where the multiple components in HEAs interact, resulting in slow atomic diffusion and hindering phase precipitation [4]; and the “cocktail” effect in properties, where the distinct physical and chemical characteristics of different atoms bring unique cocktail effects to HEAs, enabling them to possess multiple functional properties simultaneously [5].

Based on the positions of the main elements in the periodic table, HEAs are mainly classified into transition metal high-entropy alloys and refractory high-entropy alloys [6]. Transition metal high-entropy alloys, with their advantages of high specific strength and high specific hardness, hold application potential in aerospace, aviation, energy, transportation, and other fields, and have been widely studied by many scholars. However, their high-temperature mechanical properties are not satisfactory. Researchers introduced refractory metals into HEAs, forming refractory high-entropy alloys (RHEAs) with refractory metal elements as the main components. In 2010, Senkov et al. [7] developed two body-centered cubic (BCC) RHEAs, namely MoNbTaW and MoNbTaVW, and the research results indicated their excellent high-temperature mechanical properties. Subsequently, researchers have discovered refractory high-entropy alloys such as AlNbTiV [8], NbHfTiV [9], and AlCrNbTiV [10] with favorable mechanical properties. In addition, RHEAs also possess good wear resistance [11], irradiation resistance [12], and other characteristics, thus having great application potential in aerospace, electronics, chemical engineering, nuclear reactors, and other fields [13,14].

Among the discovered refractory high-entropy alloy systems, the NbZrTi-based refractory high-entropy alloys exhibit excellent mechanical properties. The NbZrTi-based refractory high-entropy alloys have broad composition and performance regulation spaces, endowing the alloys with strengthening and toughening mechanisms such as dislocation strengthening, precipitation strengthening, phase transformation strengthening, and twin-induced strengthening [15,16], thereby achieving the optimal combination of strength and plasticity through the coupling of multiple mechanisms. The NbZrTi-based refractory high-entropy alloys are generally in the BCC phase, but with the addition of different elements and their proportions, the phase composition and mechanical properties of the alloys also vary. Currently, many studies have been conducted on NbZrTi-based refractory high-entropy alloys, such as NbZrTiVAl [17], NbZrTiMoV [18], NbZrTiHfScAl [19], NbZrTiTaMo [20], etc. Among these, the NbZrTiHfMo high-entropy alloy consists of a disordered BCC solid solution [21]. At room temperature, this alloy exhibits a relatively high compressive yield strength, reaching 1719 MPa. This high-strength characteristic can be attributed to the addition of Hf and Mo elements, which cause a large atomic size mismatch and thus enhance the alloy’s strength. However, in the equiatomic NbZrTiHfMo high-entropy alloy, the content of the Mo element is relatively high, and its addition significantly increases the electronegativity difference within the alloy. The electronegativity difference is a key physical characteristic parameter affecting the plasticity of the alloy. Therefore, the high content of the Mo element leads to a decrease in the alloy’s plasticity [22]. In contrast to the equiatomic alloy, the Nb_35_Zr_26_Ti_19_Hf_15_Mo_5_ alloy developed by us exhibits a better strength–plasticity combination. Moreover, the NbZrTiHfMo high-entropy alloy in this study has not undergone rolling treatment, while rolling treatment can usually eliminate the casting defects of the alloy and improve its strength [23]. In this paper, the Nb_35_Zr_26_Ti_19_Hf_15_Mo_5_ alloy was prepared by the suspension melting method. A new understanding of the microscopic structures of the alloy in both as-cast and rolled states was obtained, and the influencing factors on the mechanical properties of this alloy were expounded.

## 2. Materials and Methods

### 2.1. Materials Preparation

The Nb_35_Zr_26_Ti_19_Hf_15_Mo_5_ (atomic percent, at.%) alloy was prepared by vacuum levitation melting using a water-cooled copper crucible in the smelting equipment SMTX-XF-10 (SUMMIT NEW MATERIALS). The furnace vacuum degree was 3 × 10^−3^ Pa, and high-purity argon gas was used for protection. The process of repeated remelting was carried out 5–6 times to ensure the homogeneity of the ingot. The cast ingot weighed 5 kg, with a height of approximately 141 mm and a diameter of about 107 mm. Since the edge part of the ingot had fewer defects, all test samples were taken from the edge part of the ingot.

Several cuboid specimens with dimensions of 60 mm × 30 mm × 10 mm were cut from the Nb_35_Zr_26_Ti_19_Hf_15_Mo_5_ alloy ingot by wire cutting method for cold rolling experiments. The multi-pass rolling method was adopted to roll in the lengthwise direction. The original thickness of the rolled sample is 10 mm. The rolling process is carried out along the length direction. The linear velocity of the rolling mill rolls is 600 mm/s, and the reduction per pass is 0.1 mm. The rolling experiment was designed with three rolling reductions, namely 20%, 50%, and 80%, and the samples corresponding to these reductions were designated as Alloy-20, Alloy-50, and Alloy-80, respectively.

### 2.2. Microstructure Characterization

Samples with dimensions of 6 mm × 6 mm × 6 mm were cut from the ingot by electric discharge machining for microstructure and phase analysis. The crystal structure was studied using an X-ray diffractometer (SmartLab, Rigaku Corporation, Akishima, Japan) with a 2θ scanning range of 20–100°, a scanning speed of 5°/min, a voltage of 40 KV, a current of 150 mA, and a Cu target. The microstructure characteristics were observed using a scanning electron microscope (Sigma-300, Zeiss, Oberkochen, Germany) and a transmission electron microscope (Tecnai G2 F20, Thermo Fisher Scientific, Waltham, MA, USA). Electron backscatter diffraction (Symmetry, Oxford, UK) was further employed to analyze the microstructure.

### 2.3. Mechanical Property Testing

Tensile tests were carried out using a universal tensile testing machine (WDW-20, Changchun Kexin Testing Machine Co., Ltd., Jilin, China). Tensile specimens were cut from the middle position of the rolled plate along the tensile direction, and the specimen surfaces were polished to be smooth. During the experiment, the tensile rate of the specimen was 1 mm/min. To reduce experimental errors, three groups of tensile tests were performed under each process parameter.

## 3. Results

### 3.1. Crystal Structure and Microstructure

**Figure 1:**
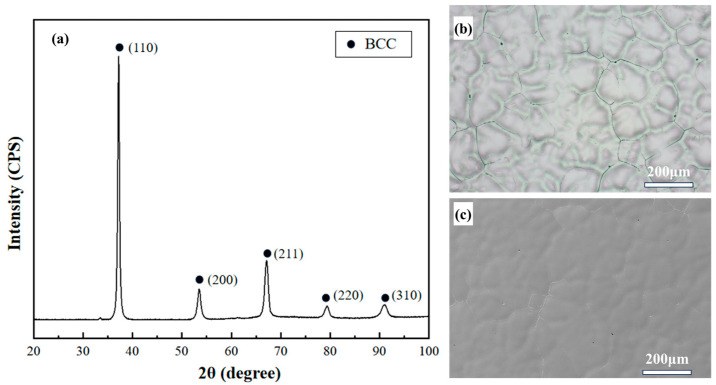
(**a**) XRD pattern of the Nb_35_Zr_26_Ti_19_Hf_15_Mo_5_ refractory high-entropy alloy; (**b**) optical microscopy microstructure image of the alloy after corrosion; (**c**) SEM microstructure image of the alloy after corrosion.

**Figure 2:**
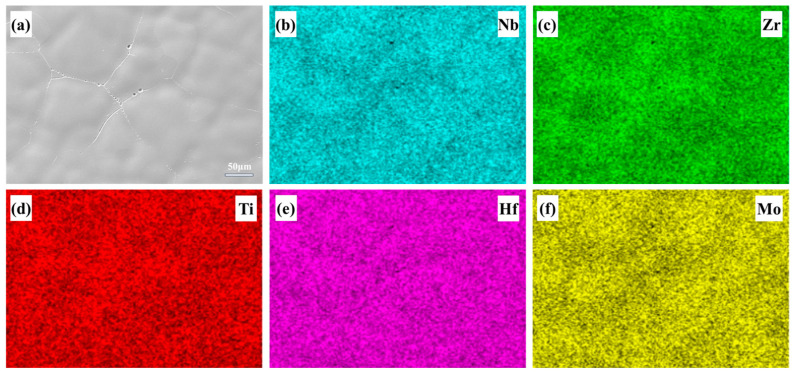
Mapping images of the Nb_35_Zr_26_Ti_19_Hf_15_Mo_5_ refractory high–entropy alloy: (**a**) original scanning image; (**b**–**f**) Mapping images of Nb, Zr, Ti, Hf and Mo.

X-ray diffraction experiments were performed on the Nb_35_Zr_26_Ti_19_Hf_15_Mo_5_ alloy. Figure 1 shows the XRD pattern of the as-cast alloy. From the XRD diffraction pattern, it can be seen that the Nb_35_Zr_26_Ti_19_Hf_15_Mo_5_ alloy consists of a single-phase BCC solid solution, and there is no ordered peak at the low-angle diffraction position. By substituting into the Bragg equation 2dsinθ = nλ, it can be calculated that the lattice constant is 3.411 Å, and the crystal planes are (110), (200), (211), (220), and (310). The microstructure of the as-cast Nb_35_Zr_26_Ti_19_Hf_15_Mo_5_ alloy is shown in Figure 1b,c. The grain size of the as-cast alloy is approximately 100 μm. After etching, a typical dendritic and interdendritic structure was observed. Figure 2 shows the EDS mapping of the alloy. It can be seen from the figure that the dendrite trunk of the alloy is enriched with high-melting-point elements such as Nb, Hf, and Mo, but the enrichment degree is relatively low, and the elemental composition difference between the dendrite trunk and the interdendritic region is small.

### 3.2. Influence of Different Reduction Amounts on the Microstructure and Mechanical Properties of Alloys

**Figure 3:**
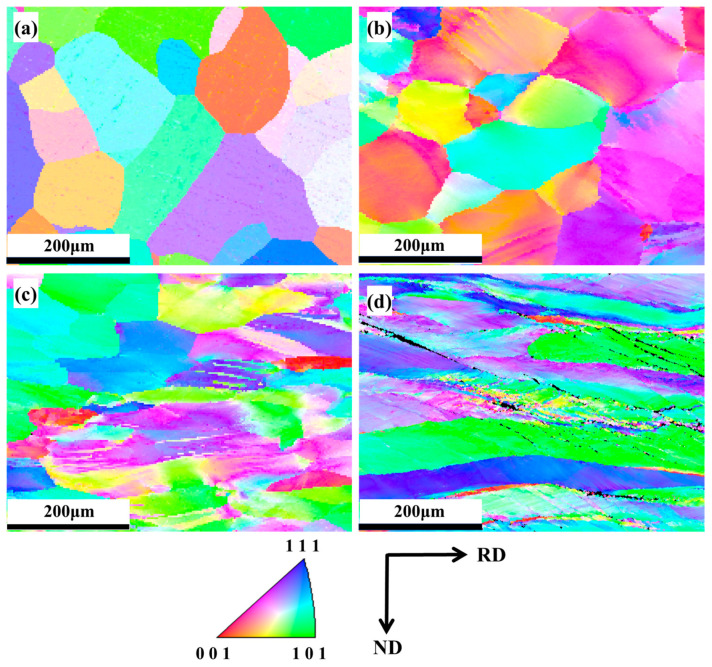
EBSD-IPF maps of the Nb_35_Zr_26_Ti_19_Hf_15_Mo_5_ refractory high-entropy alloy: (**a**) as-cast state; (**b**) 20% rolling reduction; (**c**) 50% rolling reduction; (**d**) 80% rolling reduction.

Figure 3a–d presents the EBSD-IPF maps corresponding to the as-cast state and Alloy-20, Alloy-50, and Alloy-80, respectively. It can be seen from Figure 3a that Alloy-20, fabricated through vacuum levitation melting, exhibits equiaxed grains in the as-cast state without orientation distribution characteristics. Upon 20% rolling deformation of Alloy-20, a substantial amount of equiaxed grains persists in the alloy, with a small portion of grains being elongated along the rolling direction. At this stage, no distinct orientation distribution characteristics are observable in Alloy-20. When the rolling deformation reaches 50%, a majority of the grains in Alloy-50 are deflected and elongated along the rolling direction. With a further increase in the rolling deformation to 80%, the equiaxed grains in Alloy-80 vanish, and fine recrystallized grains emerge at certain grain boundaries.

**Figure 4:**
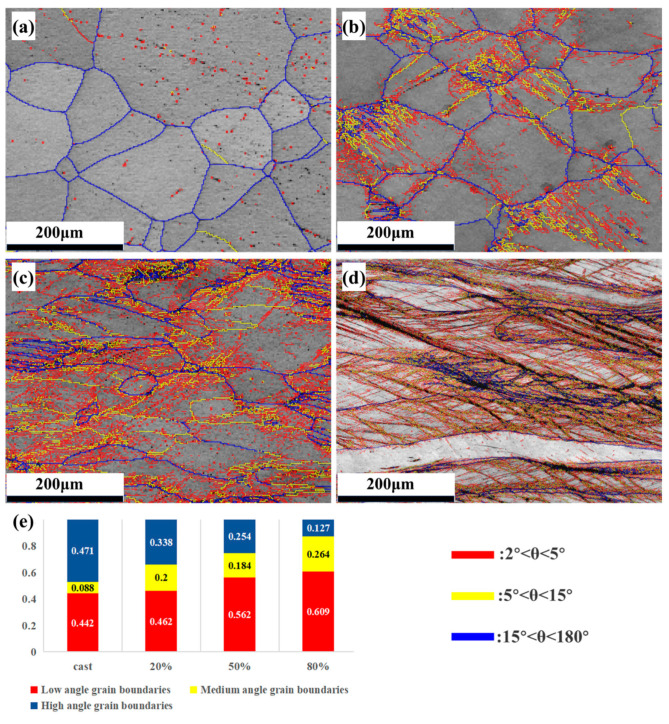
Nb_35_Zr_26_Ti_19_Hf_15_Mo_5_ refractory high-entropy alloy: (**a**) as-cast state; (**b**) 20% rolling reduction; (**c**) 50% rolling reduction; (**d**) 80% rolling reduction; (**e**) statistical chart of the proportion of high- and low-angle grain boundaries.

During the rolling deformation process, as the degree of deformation increases, the content of grain boundaries and the density of sub-structures such as dislocations in the alloy change accordingly. Figure 4a–d shows the EBSD-distribution maps of low-and high-angle grain boundaries of the Nb_35_Zr_26_Ti_19_Hf_15_Mo_5_ alloy in the as-cast state and under rolling reductions of 20%, 50%, and 80%. In Figure 4, the red lines represent low-angle grain boundaries with an orientation difference of 2–5°, the green lines represent medium-angle grain boundaries with an orientation difference of 5–15°, and the blue lines represent high-angle grain boundaries with an orientation difference of 15–180°. As can be seen from Figure 4a, in the as-cast alloy, high-angle grain boundaries are only present at the grain boundaries, and the content of low-angle grain boundaries within the grains is relatively low. However, after rolling, a large number of low- and medium-angle grain boundaries appear within the grains. As shown in Figure 4b,c, after the alloy is rolled and deformed by 20% and 50%, the grains are elongated along the rolling direction, and multiple sets of parallel high-angle twin boundaries (blue parallel line groups) appear in some grains. Between these high-angle boundaries, it is easy to accumulate some low- and medium-angle grain boundaries. When the rolling deformation of the alloy reaches 80% (Figure 4d), the density of low- and medium-angle grain boundaries further increases. With the increase in the rolling deformation degree, in the as-cast alloy, the alloy with 20% deformation, the alloy with 50% deformation, and the alloy with 80% deformation, the proportion of the content of low- and medium-angle grain boundaries is 0.530, 0.662, 0.746, and 0.849, respectively, and the overall density of grain boundaries increases.

**Figure 5:**
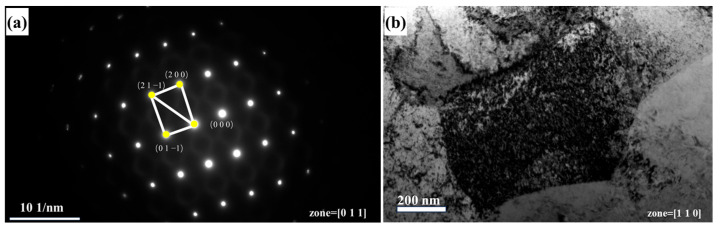
Alloy with a rolling reduction of 80%: (**a**) diffraction spot; (**b**) TEM image.

TEM analysis was conducted on the alloy with an 80% rolling reduction. Figure 5a reveals that the alloy remains in a single-phase BCC structure after rolling, without the appearance of second phases, which is consistent with the XRD results in Figure 1. In Figure 5b, a large number of dislocation agglomerations are observed in the alloy. These dislocation agglomerations can effectively impede the further movement of dislocations, thereby enhancing the strength of the alloy. Meanwhile, a relatively high amount of energy is stored at the sites of dislocation agglomerations. During subsequent heat treatment, atomic rearrangement and nucleation processes will occur preferentially in these high-energy regions, giving rise to new strain-free grains. This, in turn, promotes the evolution of the alloy’s microstructure and the refinement of grains.

**Figure 6:**
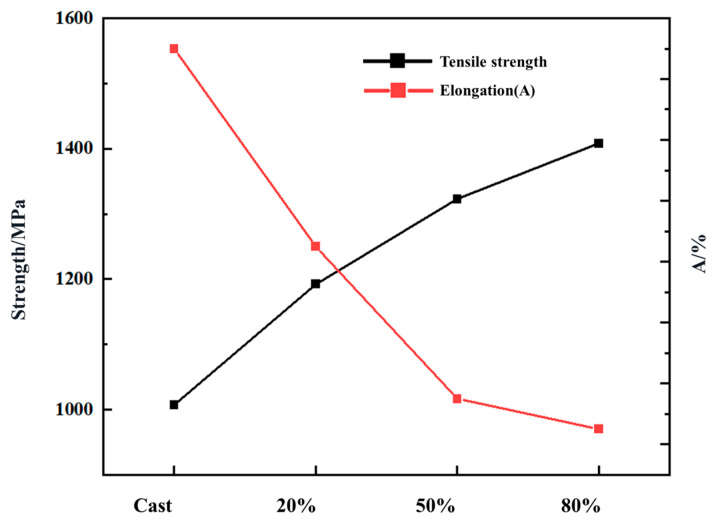
Tensile mechanical properties of the Nb_35_Zr_26_Ti_19_Hf_15_Mo_5_ refractory high-entropy alloy in the as-cast state and with rolling reductions of 20%, 50%, and 80%.

Figure 6 shows the tensile mechanical properties of the Nb_35_Zr_26_Ti_19_Hf_15_Mo_5_ refractory high-entropy alloy in the as-cast state and with rolling reductions of 20%, 50%, and 80%, revealing the variation law of the alloy mechanical properties with the deformation degree. It can be observed from the figure that the yield strength of the as-cast alloy is 562 MPa, the tensile strength is 1007 MPa, and the elongation after fracture is 23%. With the gradual increase in the rolling deformation amount, the tensile strength of the alloy significantly increases from 1007 MPa to 1408 MPa, while the elongation after fracture decreases from 23% to 10.5%, indicating that with the increase in the alloy rolling reduction, the strength increases, while the plasticity decreases.

### 3.3. Effect of Annealing Temperature on the Microstructure of the Nb_35_Zr_26_Ti_19_Hf_15_Mo_5_ Refractory High-Entropy Alloy

To eliminate the work hardening and internal stress generated during the cold working process, the Nb_35_Zr_26_Ti_19_Hf_15_Mo_5_ refractory high-entropy alloy with a rolling reduction of 80% was annealed.

**Figure 7:**
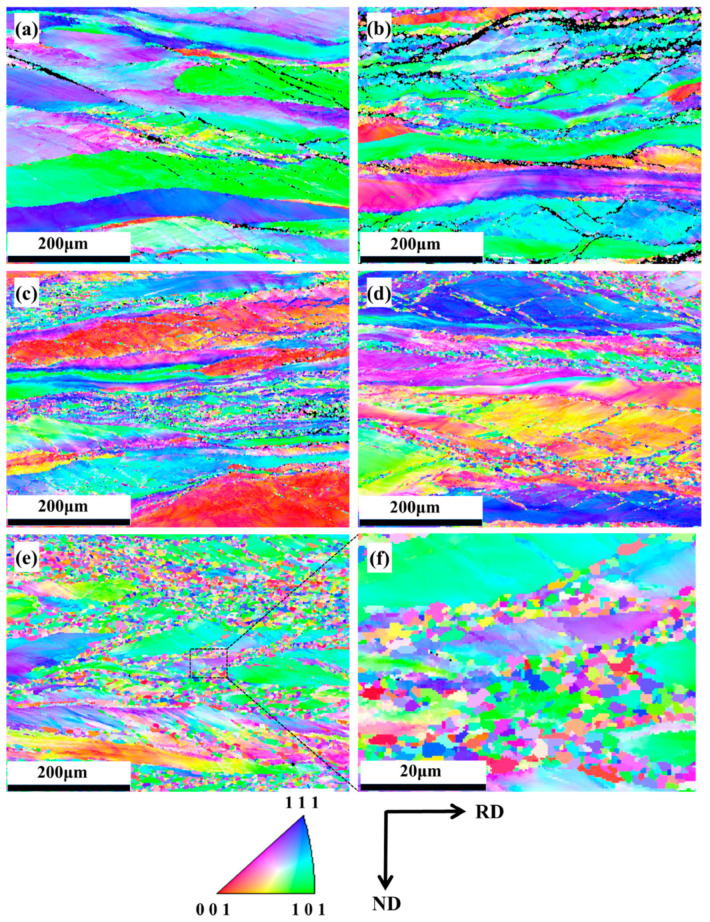
(**a**) EBSD-IPF map of the Nb_35_Zr_26_Ti_19_Hf_15_Mo_5_ refractory high-entropy alloy with a rolling reduction of 80% without heat treatment and after annealing at (**b**) 650 °C; (**c**) 700 °C; (**d**) 750 °C; (**e**,**f**) 800 °C for 20 min.

Figure 7a–e shows the EBSD-IPF maps of the Nb_35_Zr_26_Ti_19_Hf_15_Mo_5_ refractory high-entropy alloy with a rolling reduction of 80% without heat treatment and after recrystallization annealing at 650 °C, 700 °C, 750 °C, and 800 °C for 20 min, respectively. Microstructural evolution analysis reveals that the original matrix-phase grains experienced significant elongation along the rolling direction during deformation. During subsequent recrystallization, these elongated grains became progressively enveloped by newly formed recrystallized grains with homogeneous orientations, ultimately developing a distinctive necklace-like microstructure. Notably, the monotonic augmentation of fine-scale recrystallized grain density manifests as a dominant characteristic with progressive elevation of annealing temperature.

**Figure 8:**
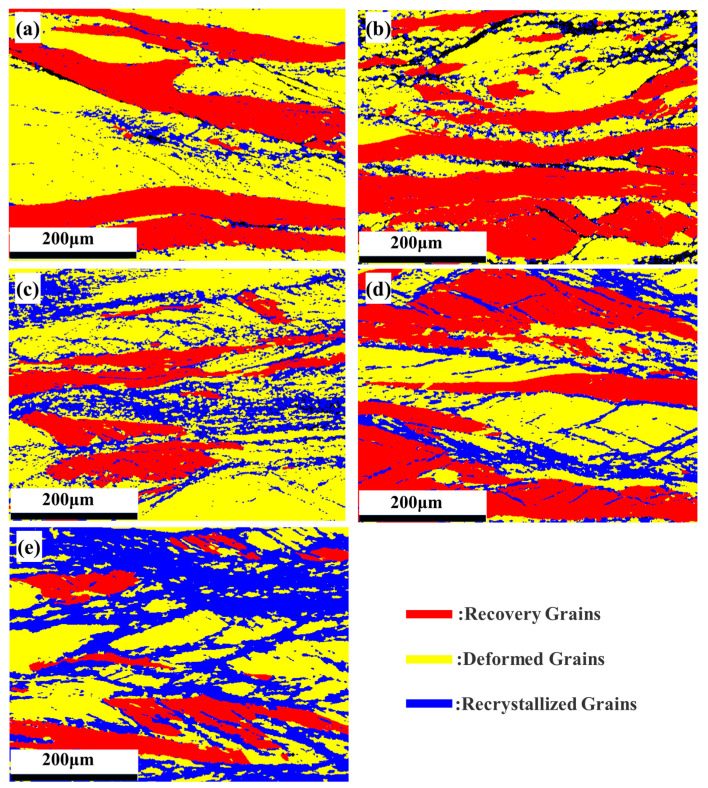
(**a**–**e**) EBSD-GOS maps of the microstructure evolution of the Nb_35_Zr_26_Ti_19_Hf_15_Mo_5_ refractory high-entropy alloy with a rolling reduction of 80% without heat treatment and after annealing at 650 °C, 700 °C, 750 °C, and 800 °C for 20 min.

**Figure 9:**
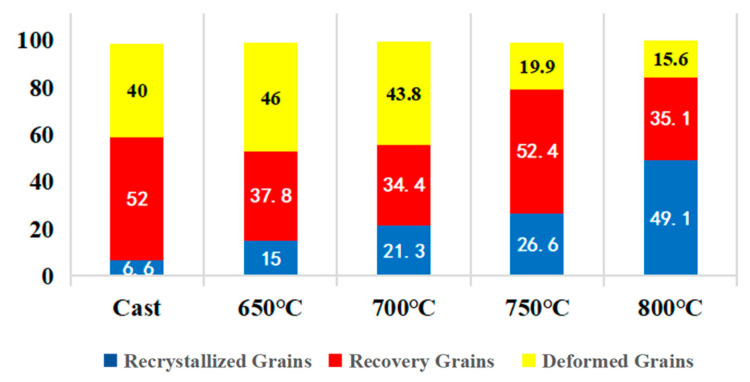
Proportion of grains in the GOS map of the Nb_35_Zr_26_Ti_19_Hf_15_Mo_5_ refractory high-entropy alloy.

Figure 8a–e presents the EBSD-GOS maps of the microstructure evolution of the Nb_35_Zr_26_Ti_19_Hf_15_Mo_5_ refractory high-entropy alloy. The alloy underwent a rolling reduction of 80% and was then subjected to different treatment conditions: without heat treatment and annealing at 650 °C, 700 °C, 750 °C, and 800 °C for 20 min. In the figure, the blue region indicates the recrystallized grains with a grain orientation difference of less than 2°, which represents the core region of recrystallization, while the red region indicates the recovery region with a grain orientation difference of between 2° and 7°, which reflects the preliminary recovery of the plastic deformation experienced by the material during the rolling process. In addition, the yellow region with an orientation difference of 7–20° indicates the deformed grains remaining after rolling, and these grains have a large orientation difference, showing traces of plastic deformation. Through observation, it is found that in the case of no annealing treatment, the microstructure of the alloy is mainly composed of deformed grains, presenting an obvious deformation texture. Figure 9 shows the proportion of each region in the GOS map. Upon annealing the alloy at 650 °C, the proportion of the recrystallization region is only 15%, indicating that the effect of annealing on promoting recrystallization is relatively weak. Subsequently, with the gradual increase in the annealing temperature, the recrystallization process is significantly accelerated. Specifically, at 700 °C, the proportion of the recrystallization region rises to 21.3% and further increases to 26.6% at 750 °C. Furthermore, when the annealing temperature reaches 800 °C, the proportion of the recrystallization region significantly increases to 49.1%, clearly indicating that the increase in the annealing temperature has a significant promoting effect on the recrystallization kinetics.

**Figure 10:**
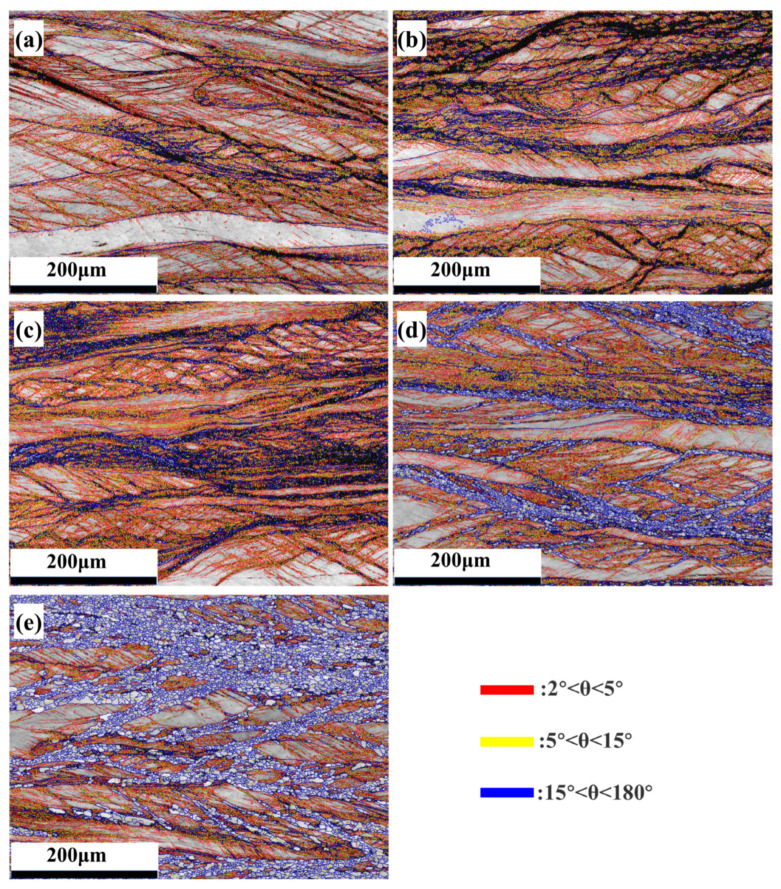
(**a**–**e**) Maps of the size and angle of the grain boundaries of the Nb_35_Zr_26_Ti_19_Hf_15_Mo_5_ refractory high-entropy alloy with a rolling reduction of 80% without heat treatment and after annealing at 650 °C, 700 °C, 750 °C, and 800 °C for 20 min.

Figure 10 shows the maps of the size and angle of the grain boundaries of the Nb_35_Zr_26_Ti_19_Hf_15_Mo_5_ refractory high-entropy alloy with a rolling reduction of 80% after annealing at different temperatures (650 °C, 700 °C, 750 °C, and 800 °C) for 20 min. These maps provide crucial insights into the microstructural evolution of the alloy during the annealing process.

During annealing, thermal energy is imparted to the alloy, which promotes atomic diffusion and rearrangement within the crystal lattice. As the annealing temperature rises, the content of grain boundaries and the density of sub-structures such as dislocations in the alloy change significantly. At lower annealing temperatures, the atoms have limited mobility. The high amount of stored energy from the prior 80% rolling reduction maintains a relatively high density of dislocations and a large proportion of low- and medium-angle grain boundaries. These low- and medium-angle grain boundaries are formed as a result of the accumulation of dislocations during the rolling process.

In more detail, low-angle grain boundaries (LAGBs, typically with misorientation angles between 2° and 15°) are composed of arrays of dislocations. Medium-angle grain boundaries, with misorientation angles slightly higher than LAGBs, also have a relatively ordered dislocation structure. These boundaries act as barriers to dislocation motion, contributing to the strengthening of the alloy through the mechanisms of interface strengthening and dislocation strengthening.

**Figure 11:**
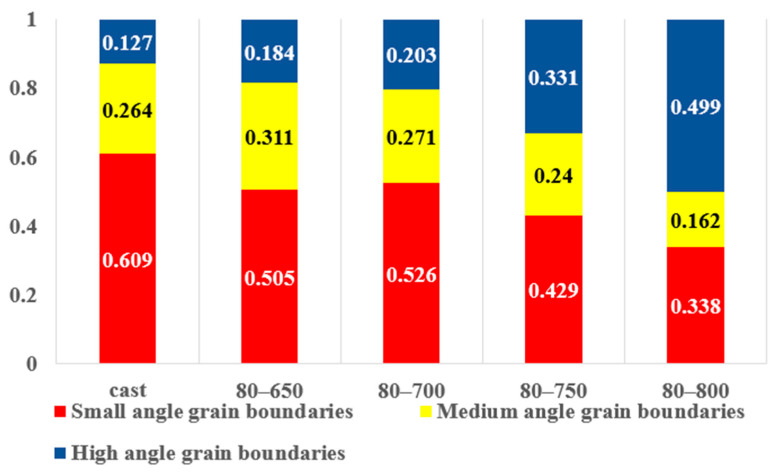
Statistical chart of the size and angle of the grain boundaries of the Nb_35_Zr_26_Ti_19_Hf_15_Mo_5_ refractory high-entropy alloy with a rolling reduction of 80% without heat treatment and after annealing at 650 °C, 700 °C, 750 °C, and 800 °C for 20 min.

As we move from lower annealing temperatures to higher ones, a variety of microstructural changes occur. An examination of Figure 10 and Figure 11 reveals a clear trend: with the increase in the annealing temperature, the proportion of low- and medium-angle grain boundaries in the alloy gradually decreases, while the proportion of high-angle grain boundaries (HAGBs, misorientation angles > 15°) increases. This phenomenon can be attributed to the enhanced atomic diffusion at higher temperatures.

The high thermal energy at elevated temperatures enables dislocations to move more freely, leading to their annihilation and rearrangement. As a result, the low- and medium-angle grain boundaries gradually transform into high-angle grain boundaries through processes such as recovery and recrystallization. Recovery involves the rearrangement and annihilation of dislocations within the existing grains, reducing the density of sub-structures. Recrystallization occurs when new strain-free grains nucleate and grow, consuming the deformed regions and forming high-angle grain boundaries.

Moreover, the overall grain boundary density decreases as the annealing temperature increases. Grain boundary density is a measure of the total length of grain boundaries per unit area. With the growth of grains during the transformation from low/medium-angle to high-angle grain boundaries and the coarsening of the microstructure, the total length of grain boundaries per unit area reduces.

This microstructural evolution has a profound impact on the mechanical properties of the alloy. The interface strengthening effect and dislocation strengthening effect in the alloy are closely related to the density and type of grain boundaries. Since low- and medium-angle grain boundaries are more effective in impeding dislocation motion compared to high-angle grain boundaries, the decrease in their proportion and the overall grain boundary density lead to a reduction in the strength of the alloy. Conversely, the formation of larger, well-defined grains and the reduction of internal lattice defects facilitate dislocation glide, resulting in an increase in the plasticity of the alloy.

In conclusion, the microstructural changes observed in Figure 10 and Figure 11, along with the corresponding variations in mechanical properties, are a direct consequence of the thermally activated processes during the annealing of the rolled Nb_35_Zr_26_Ti_19_Hf_15_Mo_5_ refractory high-entropy alloy.

**Figure 12:**
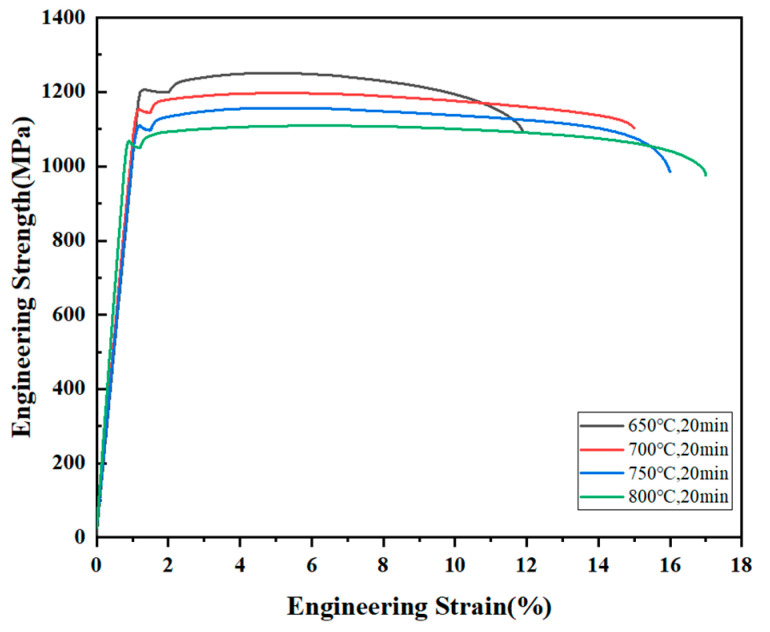
Stress–strain curves of the Nb_35_Zr_26_Ti_19_Hf_15_Mo_5_ refractory high-entropy alloy with a rolling reduction of 80% at different annealing temperatures.

The tensile mechanical properties of the tensile samples at four different temperature gradients were tested. By analyzing the stress–strain curves shown in Figure 12, the following phenomena can be observed: with the increase in the annealing temperature from 650 °C to 800 °C, the tensile strength of the Nb_35_Zr_26_Ti_19_Hf_15_Mo_5_ refractory high-entropy alloy decreases from 1251 MPa to 1069 MPa.

In terms of plasticity, the plasticity of the alloy increases with the increase in the annealing temperature. The plasticity of the alloy annealed at 650 °C is the worst, with a strain of only 11%, while the elongation after fracture of the alloy reaches a peak value of 17% at 800 °C, indicating that the plasticity of the alloy is the best. Such a result, in conjunction with the aforementioned EBSD-GOS map, suggests that at the annealing temperatures of 750 °C and 800 °C, the Nb_35_Zr_26_Ti_19_Hf_15_Mo_5_ refractory high-entropy alloy exhibits a relatively high recrystallization degree, thereby significantly improving the plasticity of the alloy.

**Figure 13:**
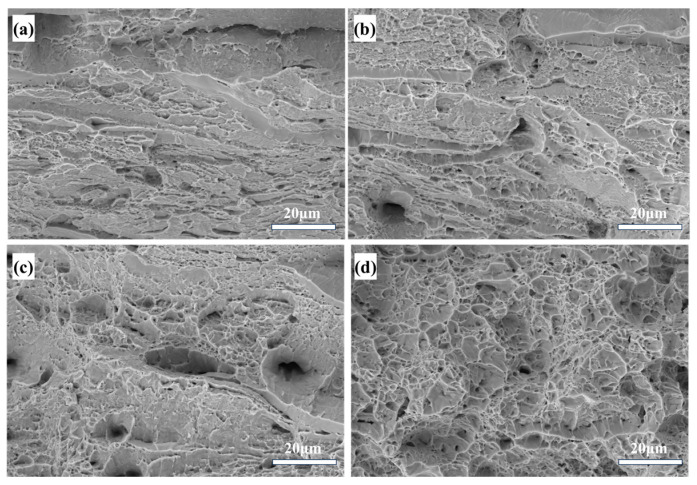
Tensile fracture morphologies of the Nb_35_Zr_26_Ti_19_Hf_15_Mo_5_ refractory high-entropy alloy with a rolling reduction of 80% at different annealing temperatures: (**a**) 650 °C, 20 min; (**b**) 700 °C, 20 min; (**c**) 750 °C, 20 min; (**d**) 800 °C, 20 min.

The fracture morphologies at different annealing temperatures were observed to further understand the change of the Alloy-80 plasticity, as shown in Figure 13. A layered structure parallel to the rolling direction can be clearly observed at the fracture for the alloys annealed at 650 °C and 700 °C. Under the annealing condition of 650 °C, there are basically no dimples at the fracture, and a river-like tearing pattern appears, showing poor plasticity and presenting the characteristics of quasi-cleavage fracture. When the annealing temperature is increased to 700 °C, micropores begin to appear in the fracture, indicating an increase in plasticity. Under the annealing conditions of 750 °C and 800 °C, the alloy has good plasticity and a relatively high recrystallization degree, and the number of dimples in the fracture increases. There is a direct link between recrystallization and dimple formation. During dynamic recrystallization, small grains are formed, increasing the number of grain boundaries. Since vacancies tend to accumulate at grain boundaries, their aggregation eventually leads to the formation of more dimples at these boundaries, explaining the presence of more dimples in dynamically recrystallized areas.

## 4. Discussion

### 4.1. Formation Mechanism of Microstructure

#### 4.1.1. Analysis of the Formation Mechanism of the BCC Phase

The phase formation of high-entropy alloys is determined by multiple factors. In the study by Y. Zhang et al. [24], it was found that phase formation in high-entropy alloys is a complex process involving the interaction of multiple factors. High-entropy alloys contain multiple elements and are mixed in an equal or near-equal molar ratio. This complex composition combination makes the phase formation mechanism not simply attributable to a single factor. Accordingly, Y. Zhang et al. proposed Equation (1):

(1) $$\Omega =T_{m}{\Delta S}_{mix}/|{\Delta H}_{mix}|$$

Based on the calculation formulas of mixing entropy and mixing enthalpy as well as the data in Table 1 (the values of pairwise element mixing enthalpy) [21], the ${\Delta S}_{mix}$ of this alloy can be calculated as 12.55 J/mol, and the ${\Delta H}_{mix}$ is 1824 J/mol. Further calculation yields Ω=14.64, that is, Ω≫1, indicating a high probability of the formation of a solid solution in this high-entropy alloy.

The valence electron concentration, defined as the ratio of the total number of valence electrons of each constituent element in the alloy to the total number of atoms, significantly influences the solid-solution stability and is closely related to the formation of alloy phases [25]. Guo et al. [26], based on the valence electron concentration theory, analyzed and organized the stabilities of the FCC (face-centered cubic) and BCC (body-centered cubic) phases during high-entropy alloy formation. They discovered that the VEC (valence electron concentration) can quantitatively predict the phase stabilities of the FCC and BCC phases in HEAs (high-entropy alloys). For VEC ≥ 8.0, only the FCC phase exists; in the range of 6.87 < VEC < 8.0, both FCC and BCC phases co-exist; and when VEC ≤ 6.87, only the BCC phase is present. Zhang et al. [27], in line with the “Hume-Rothery criterion”, found that an alloy with an atomic radius difference of less than 6.5% has a greater tendency to form a single-phase solid solution. The Nb_35_Zr_26_Ti_19_Hf_15_Mo_5_ alloy, with a VEC of 4.45 and an atomic radius difference of 4.5%, forms a single-phase BCC structure.

#### 4.1.2. Microstructure Evolution During Rolling and Heat Treatment

In the study of rolling the Nb_35_Zr_26_Ti_19_Hf_15_Mo_5_ refractory high-entropy alloy, it was observed that in the as-cast state, the alloy exhibited an isotropic equiaxed grain morphology, which originated from the uniform heat dissipation during solidification, leading to the random arrangement and aggregation of atoms. As the rolling process progressed, upon reaching a 20% rolling reduction, a portion of the grains was affected by stress and elongated along the rolling direction due to the initiation of dislocation slip within the crystal under stress. With the rolling reduction increasing to 50%, most of the grains were significantly deformed, and dislocations accumulated and entangled at the grain boundaries, storing energy for subsequent structural transformation. When the rolling reduction reached 80%, the equiaxed grains disappeared, and a textured structure with a preferred orientation was formed as the crystal orientations tended to align to adapt to the rolling force. Meanwhile, in regions with a high dislocation density where deformation energy storage was excessive, recrystallized grains emerged at certain grain boundaries.

Upon annealing the alloy that has undergone an 80% rolling reduction, significant changes occur in the microstructure. With the increase in annealing temperature, the recrystallized grains gradually increase. At 650 °C, the recrystallized region constitutes 15% of the total area, and it significantly increases to 49.1% at 800 °C. The recrystallization kinetics were analyzed based on the Avrami equation [28]:

X = 1 − exp (−kt^n^) (2)

where X represents the recrystallized fraction, t denotes the annealing time, k is the recrystallization rate constant, and *n* refers to the Avrami exponent. The calculated results demonstrate that the rate constant k increases significantly with elevated annealing temperatures, exhibiting values of 1.05 × 10^−4^  min^−1.5^ at 650 °C and 1.22 × 10^−3^  min^−1.5^ at 800 °C. This trend evidently demonstrates that elevating the annealing temperature exerts a remarkable promoting influence on the recrystallization process. For the recrystallization process, the Arrhenius equation can be equivalent to the following [29]:

G = Ate^−Q/RT^ (3)

In the formula, G is the volume fraction of the recrystallized grains; A is a constant; t is the annealing time; Q is the recrystallization activation energy, which is a fixed value when the material composition and processing technology remain unchanged; R is the gas constant; and T is the annealing temperature. According to this formula, the activation energy Q can be calculated to be 103.9 kJ/mol. The increase in the annealing temperature has a significant promoting effect on recrystallization. The reason is that with the increase in annealing temperature, the atomic diffusion ability is enhanced, which helps to accelerate the movement and recombination of dislocations, thereby promoting the release of deformation energy storage. In addition, the increase in temperature reduces the energy barrier for recrystallization nucleation, enabling more deformation energy storage to be converted into the driving force for recrystallization, thereby increasing the recrystallization nucleation rate and the growth rate of grains.

### 4.2. Analysis of the Mechanical Properties of the Alloy

The Nb_35_Zr_26_Ti_19_Hf_15_Mo_5_ alloy has a single-phase BCC structure, so solid solution strengthening and dislocation strengthening are the main sources of the alloy’s strength.

#### 4.2.1. Solid Solution Strengthening

The Nb_35_Zr_26_Ti_19_Hf_15_Mo_5_ alloy has a single-phase BCC structure, so solid solution strengthening is an important strengthening mechanism [30]. The main elements of the high-entropy alloy randomly occupy different positions in the lattice, and there is no distinction between solute and solvent atoms. This random and disordered occupation makes the atomic occupation around each atom different. Because the atomic sizes and moduli of each element are different, strong size mismatch and modulus mismatch effects are produced in the crystal structure, resulting in an increase in lattice distortion. Around small-sized atoms, the crystal structure collapses relative to other positions, and near large-sized atoms, the crystal structure expands compared to other positions. In addition, the chemical bonding between different atoms and the surrounding atoms is also different, resulting in a modulus difference at different positions in the crystal structure. When the dislocation motion encounters this stress field, an interaction occurs, hindering the dislocation motion and thus producing a solid solution strengthening effect. After considering the size difference and modulus difference among different elements, the influences of element i on the size mismatch and modulus mismatch of the solid solution are $\delta r_{i}$ and $\delta G_{i}$, respectively, which are expressed by Equations (4) and (5) [31]:

(4) $$\delta r_{i}=\frac{\delta r_{\mathrm{ijklm}}^{\mathrm{ave}}-\;\delta r_{\mathrm{jklm}}^{\mathrm{ave}}}{\delta c_{i}}$$

(5) $$\delta G_{i}=\frac{\delta G_{\mathrm{ijklm}}^{\mathrm{ave}}-\;\delta G_{\mathrm{jklm}}^{\mathrm{ave}}}{\delta c_{i}}$$

$\delta r_{\mathrm{ijklm}}^{\mathrm{ave}}$ and $\delta r_{\mathrm{jklm}}^{\mathrm{ave}}$ represent the average atomic radii of quinary HEAs (ijklm) and quaternary HEAs (jklm), respectively; $\delta G_{\mathrm{ijklm}}^{\mathrm{ave}}$ and $\delta G_{\mathrm{jklm}}^{\mathrm{ave}}$ represent the average moduli of quinary HEAs (ijklm) and quaternary HEAs (jklm), respectively. $\delta c_{i}$ represents the difference in atomic percentage of element i in HEAs (ijklm) and HEAs (jklm). Since HEAs (jklm) does not contain element i, the atomic percentage of element i is 0.

**Figure 14:**
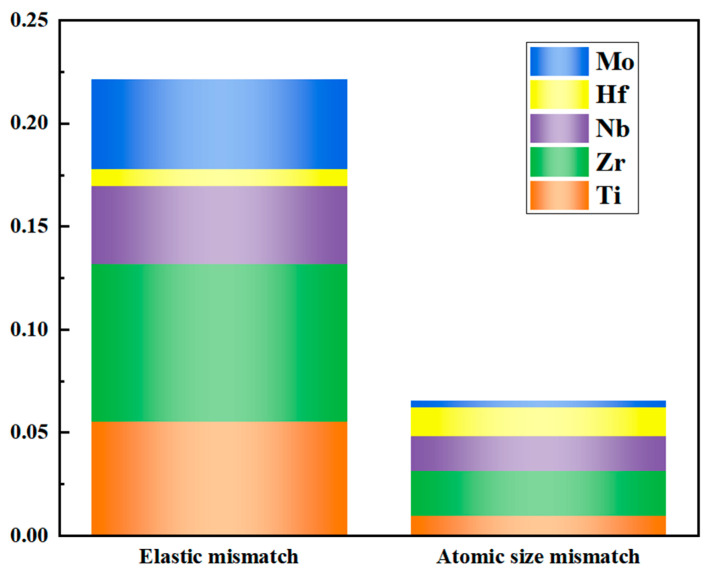
Misfit degree of the Nb_35_Zr_26_Ti_19_Hf_15_Mo_5_ refractory high-entropy alloy.

The calculation results of the misfit degree of the Nb_35_Zr_26_Ti_19_Hf_15_Mo_5_ alloy are shown in Figure 14. The solid solution strengthening effect of the alloy is closely related to the misfit degree. The larger the misfit degree, the better the solid solution strengthening effect of the alloy. Based on the comprehensive calculation results of the misfit degree, the following conclusions can be drawn: the contributions of the Zr element to the modulus misfit degree and the atomic size misfit degree are 34% and 33%, respectively. It plays a major role in solid solution strengthening, which is related to the large difference in its atomic size from other elements. It is worth noting that although the addition amount of the Mo element is only 5%, its contribution to the modulus misfit degree reaches 20%, having a relatively large influence.

#### 4.2.2. Texture

**Figure 15:**
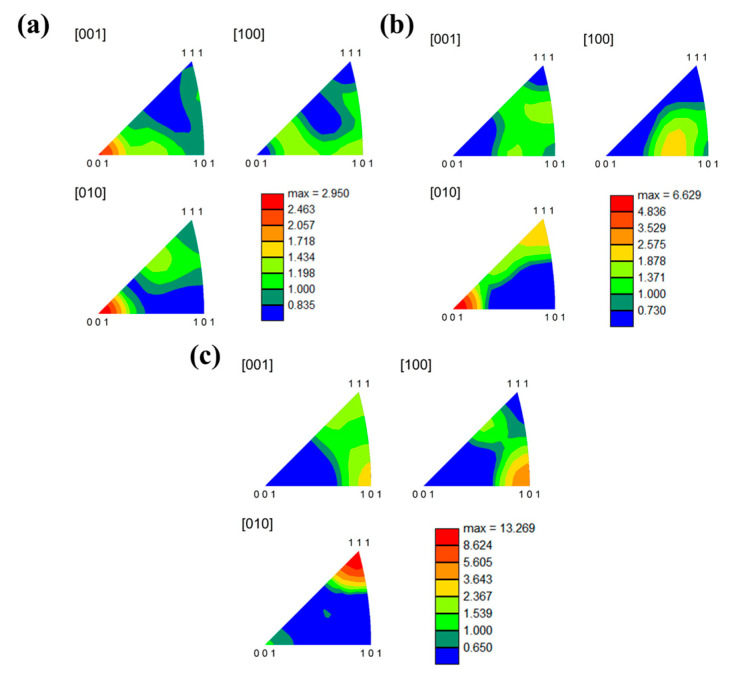
EBSD-IPF diagrams of the Nb_35_Zr_26_Ti_19_Hf_15_Mo_5_ alloy under different rolling reductions: (**a**) 20%; (**b**) 50%; (**c**) 80%.

As the rolling reduction increases, the texture strength in the Nb_35_Zr_26_Ti_19_Hf_15_Mo_5_ alloy is shown in Figure 15. It can be observed from Figure 15c that an obvious <101> (111) texture is formed in the alloy with a rolling reduction of 80%, and the texture strength reaches 13.269. Therefore, it can be seen that texture is also one of the important reasons for enhancing the strength of the alloy.

#### 4.2.3. Dislocation Strengthening

During the cold rolling process, with the increase in the rolling reduction amount, the tensile strength of the alloy increases, and with the increase in the annealing temperature, the tensile strength of the alloy decreases. This is attributed to the change in the dislocation density during the rolling process. When the material undergoes plastic deformation, the dislocations aggregate through shear slip motion to form a dislocation network, which in turn hinders the dislocation motion, produces work hardening, and increases the strength of the metal material.

Figure 4 and Figure 10 illustrate the high- and low-angle grain boundary diagrams of the alloy. It can be seen from the figures that after rolling, the number of low-angle grain boundaries in the alloy increases. This is because a large number of dislocations are arranged to form dislocation walls in the alloy after rolling, increasing the strength of the alloy. With the increase in heat-treatment temperature, the number of low-angle grain boundaries in the alloy decreases. This is because heat treatment leads to the recombination and cancellation of dislocations and a decrease in dislocation density, reducing the strength of the alloy.

## 5. Conclusions

This study systematically investigates the phase formation principles and mechanical properties of the Nb_35_Zr_26_Ti_19_Hf_15_Mo_5_ refractory high-entropy alloy (RHEA), revealing the following critical characteristics:

(1) The as-cast Nb_35_Zr_26_Ti_19_Hf_15_Mo_5_ RHEA exhibits a single-phase body-centered cubic (BCC) solid solution structure with exceptional phase stability. The significant atomic radius disparity of Zr predominantly contributes to solid solution strengthening, endowing the alloy with a tensile strength of 1007 MPa and an elongation of 23%, demonstrating remarkable strength–ductility synergy.
(2) Cold rolling substantially modifies the microstructure and mechanical behavior: at 80% rolling reduction, the dislocation density increases by a factor of 3.2 compared to the as-cast state, while the proportion of low-angle grain boundaries (LAGBs) rises from 53.0% to 84.9%. These microstructural changes elevate the tensile strength to 1408 MPa (a 40% enhancement), albeit with reduced elongation (10.5%). This strength–ductility trade-off is attributed to the combined effects of work-hardening and dislocation-blocking mechanisms.
(3) As regards strengthening mechanisms, the alloy’s mechanical strengthening arises from two synergistic mechanisms. The first is solid solution strengthening, which is dominated by atomic size mismatch (33% contribution from Zr) and modulus disparity (34% contribution from Zr). The second is dislocation strengthening, which is achieved through dislocation tangles and LAGBs generated during rolling processes, effectively hindering dislocation motion.
(4) Systematic investigations will be carried out to conduct full recrystallization annealing at elevated temperatures (>800 °C) to elucidate the microstructure evolution mechanisms. High-temperature tensile testing (≥800 °C) will be carried out to systematically evaluate the mechanical performance evolution and deformation mechanisms under thermal service conditions.

## Figures and Tables

**Table 1:** Enthalpy change values of pairwise element mixing (kJ/mol).

Element	Nb	Zr	Ti	Hf	Mo
**Nb**	0	4	2	4	−6
**Zr**		0	0	0	−6
**Ti**			0	0	−4
**Hf**				0	−4
**Mo**					0

## Data Availability

All data are available from the corresponding author upon reasonable request.
